# Thick Glass High-Quality Cutting by Ultrafast Laser Bessel Beam Perforation-Assisted Separation

**DOI:** 10.3390/mi15070854

**Published:** 2024-06-29

**Authors:** Suwan Chen, Yuxuan Luo, Xinhu Fan, Congyi Wu, Guojun Zhang, Yu Huang, Youmin Rong, Long Chen

**Affiliations:** 1 State Key Laboratory of Intelligent Manufacturing Equipment and Technology, Huazhong University of Science and Technology, Wuhan 430074, China; 2 School of Mechanical Science and Engineering, Huazhong University of Science and Technology, Wuhan 430074, China

**Keywords:** thick glass, ultrafast laser, Bessel beam, laser perforation, glass cutting

## Abstract

The cutting of thick glass is extensively employed in aerospace, optical, and other fields. Although ultrafast laser Bessel beams are heavily used for glass cutting, the cutting thickness and cutting quality need to be further improved. In this research, the high-quality cutting of thick glass was realized for the first time using ultrafast laser perforation assisted by CO_2_ laser separation. Initially, an infrared picosecond laser Bessel beam was employed to ablate the soda-lime glass and generate a perforated structure. Subsequently, a CO_2_ laser was employed to induce crack propagation along the path of the perforated structure, resulting in the separation of the glass. This study investigates the influence of hole spacing, pulse energy, and the defocusing distance of the picosecond laser Bessel beam on the average surface roughness of the glass sample cutting surface. The optimal combination of cutting parameters for 6 mm thick glass results in a minimum surface roughness of 343 nm in the cross-section.

## 1. Introduction

Thick glass has gained significant popularity in aerospace [1], optical [2], and other fields due to its exceptional optical, mechanical, physical, and chemical properties [3]. The cutting quality of thick glass is insufficient to meet the manufacturing requirements. Traditional glass-cutting methods mainly include diamond wheel cutting [4] and abrasive water jet cutting [5], both of which result in unsatisfactory surface finish and significant chipping along the cutting edge due to mechanical contact [6]. In recent years, the cutting process using ultrafast lasers has received significant attention and application. This is primarily attributed to the unique and significant advantages it provides, such as high cutting accuracy, a minimal heat-affected zone, and the elimination of post-processing requirements [7].

The extensive utilization of ultrafast laser Gaussian beam ablation has been effective in achieving the high-quality cutting of glass materials [8]. However, this technique has several limitations. The energy deposition length of a Gaussian beam is relatively short, leading to a low material removal rate in a single scan. Consequently, multiple scans are necessary to complete the cutting process [9]. The repetitive ablation induced by the ultrafast laser can result in adverse side effects, such as the formation of microcracks, chipping, heat-affected zones, and rear-side ablation on the glass-cutting samples. Addressing these issues necessitates the implementation of additional measures to improve the cutting quality [10,11]. During the processing, the ultrafast Gaussian beam tends to generate V-shaped grooves. When the laser beam from the subsequent scan encounters the V-shaped ablation groove, it refracts into the glass, resulting in the formation of subsurface damage. The implementation of down-top cutting processes can mitigate this phenomenon [12]. In summary, the challenges persistently associated with ultrafast Gaussian beam ablation include a low material removal rate, tapered edges, heat accumulation from repeated scanning, and defects resulting from heat accumulation, rendering it unsuitable for cutting thick glass.

The Bessel beam provides significant advantages over the Gaussian beam for cutting thick glass, primarily because of its extended energy deposition length. Liao et al. [13] proposed a method for the full ablation and stealth cutting of 500 μm silica glass using a picosecond laser Bessel beam. The researchers investigated the effects of these parameters by adjusting the process parameters and achieved high-quality roughness in the cross-section. Feuer et al. [14] successfully cut soda-lime glass with a thickness of 8 mm by utilizing single laser etching with a picosecond pulsed Bessel beam, operating at a repetition rate of 300 kHz and a pulse energy of 2.6 mJ. However, due to the heat accumulation effect caused by high overlap, this method led to the generation of chippings and microcracks on the glass, resulting in significant damage. To address these challenges, Meyer et al. [15] developed a Bessel beam shaper for cutting thick glass. Following the stealth cutting process, external stress was applied to achieve the complete cutting and separation of glass with thicknesses ranging from 3 mm to 10 mm. It is worth noting that accuracy, efficiency, and quality can be further improved by optimizing the Bessel beam or process method [16,17,18,19,20]. A better cutting surface quality can be achieved by applying thermal stress [21,22], and carbon dioxide laser scanning is a viable option for applying thermal stress [23]. Although it is possible to achieve high cutting quality for thick glass, further research is needed to thoroughly investigate the influence and selection methods of processing parameters when utilizing ultrafast Bessel beams for cutting thick glass. Further clarification is needed regarding the principles that govern these parameters.

This study conducted an experimental investigation to determine the optimal processing parameters for cutting thick glass with a picosecond Bessel beam. Following the perforation of the glass with the picosecond Bessel beam, a CO_2_ laser scanning technique was utilized to separate the thick glass. This study aimed to investigate how the hole spacing, laser power, and defocusing distance of the picosecond Bessel beam affect the average surface roughness of the cross-sections. Through the analysis of the experimental results, this study identified the optimal combination of parameters that achieve both high efficiency and the high-quality separation of thick glass. The findings of this study highlight the potential of this method for future industrial applications. This approach offers a promising method for achieving efficient and precise cutting of thick glass, with implications for various industries.

## 2. Experiment and Methods

### 2.1. Experimental Setup

**Figure 1:**
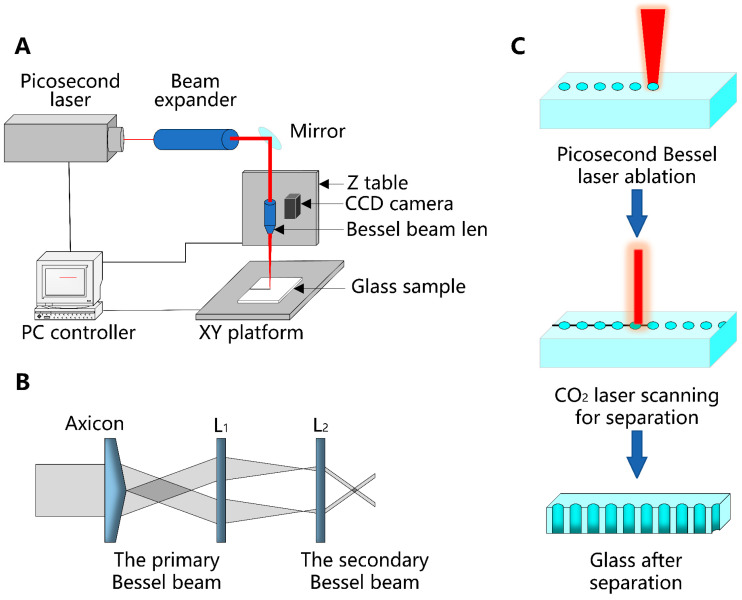
Schematic diagrams of the experimental setup for picosecond laser Bessel beam ablation: (**A**) schematic diagram of the light path and devices, (**B**) schematic diagram of the Bessel beam generation, and (**C**) glass crack propagation control and separation method.

The experiments in this study utilized a high repetition rate picosecond laser system (Amber NX-S IR-100S, Bellin Laser, Suzhou, China). The laser system has a pulse duration of 15 ps. The maximum achievable average laser power was 70 W, and the maximum pulse energy reached 1.4 mJ at a repetition rate of 50 kHz. A beam expander was utilized to adjust the spot diameter. Figure 1A illustrates the schematic diagram of the picosecond Bessel beam path. The experimental setup for generating the picosecond Bessel beam utilized an Axicon lens (EC-18, EVEOPTICS, Shanghai, China). The lens-shaped laser beam has a diameter of 4 μm and a Bessel zone of 8 mm. Figure 1B depicts the axicon-based Bessel beam generation. Soda-lime glass samples measuring 100 mm × 100 mm × 6 mm were utilized for the experiments. Glass separation in the cutting direction was performed using a CO_2_ laser with a maximum power of 400 W. Subsequently, the glass samples could be easily separated by scanning them with the CO_2_ laser after the picosecond laser perforation process. The parameters of the CO_2_ laser, including power and scanning speed, could be adjusted extensively without causing noticeable defects in the glass. The flexibility in selecting CO_2_ laser parameter selection enabled process optimization in the picosecond Bessel laser hole-drilling technique. Figure 1C illustrates the complete process of the method, encompassing picosecond laser perforation and CO_2_ laser scanning for separation.

The laser source utilized in the experiment served two crucial functions: Position Synchronized Output (PSO) and burst mode. The PSO function was employed to maintain a constant distance between micro holes throughout the scanning process. It ensured that the pulses were consistently triggered at a specific distance based on position feedback, irrespective of the stage of motion (acceleration, uniform motion, or deceleration). The repetition rate could be dynamically changed in real time by adjusting the hole spacing and scanning speed. On the other hand, the burst mode is a pulse train output mode. In burst mode, a single pulse is divided into multiple high-frequency sub-pulses, all with the same repetition frequency [24]. The total energy of the sub-pulses within a single pulse is fixed and determined by both the laser power and the repetition rate. The time interval between adjacent pulses is determined by the repetition rate, which is set by the laser source. Both the PSO and burst modes were employed to generate uniform holes in the glass with consistent spacing.

An ultra-depth microscope (VHK-7000, KEYENCE, Osaka, Japan) with deep synthesis capability was utilized to observe the cutting edges and measure the average surface roughness of the cross-sections. The morphologies of the cross-sections of the glass-cutting samples were observed using a scanning electron microscope (SU3900, HITACHI, Tokyo, Japan).

### 2.2. Method

This study utilized a combination of picosecond laser Bessel beam ablation and CO_2_ laser-controlled crack propagation to cut soda-lime glass with a thickness of 6 mm. In the initial step, a tightly arranged structure of ablating holes was created in the glass using the picosecond Bessel beam. The average diameter of the holes on the front side was measured to be 3.73 μm, which was very close to the diameter of the laser beam. In the experiments, the picosecond laser was scanned at a speed of 200 mm/s, and the repetition rate was determined based on the hole spacing and the Position Synchronized Output (PSO) function. The propagation of cracks along the direction of the micro holes could be controlled by selecting an appropriate range of micro hole spacings. In the case of thin glass, when the picosecond laser had sufficient perforation capability, and the hole spacing was appropriate, the separation of the thin glass could be achieved without the application of external stress. However, in the case of thicker glass, the cutting process could not be completed solely by picosecond laser ablation. Additional processing methods, such as inducing mechanical fatigue, were necessary to facilitate separation, albeit with potential negative effects on the cutting quality of the glass [15]. In comparison, CO_2_ laser scanning proved to be more effective in achieving glass separation and obtaining a high-quality cutting surface [25]. The scanning speed of the CO_2_ laser was set at 200 mm/s to match the scanning speed of the picosecond laser. The working distance between the CO_2_ laser lens and the glass sample was approximately 80 mm.

The described method involves applying low-power density defocused CO_2_ laser irradiation onto the glass surface. This irradiation raises the temperature of the scanning area without inducing material melting. Consequently, the glass experiences expansion due to thermal stress, which is confined by the low-temperature glass in the vicinity. This leads to the generation of compressive stress at the scanning area, which does not harm the glass. Subsequently, the temperature rapidly decreases as the laser moves away from the scanning area. As a result, the temperature differential causes the glass in that region to contract, leading to the development of tensile stress at the scanning area. Exceeding a critical value, the tensile stress leads to the formation of cracks around the laser scanning path. In the case of a single piece of intact glass, the tensile stress must surpass the crack propagation limit for cracks to occur and propagate uncontrollably. Nevertheless, when dealing with glass that possesses a perforation structure produced by picosecond Bessel beam ablation, the crack propagation limit is comparatively low, enabling controlled crack propagation. During the CO_2_ laser scanning along the micro hole path, the crack initiates from the edge of the preceding hole and halts at the edge of the subsequent hole until the last hole is reached. This controlled crack propagation facilitates the separation of the glass along the scanning path, thereby achieving the intended separation process.

This paper presents optimization experiments aimed at investigating the impact of different processing parameters on the cutting quality of the glass. The parameters examined included hole spacing, pulse energy, and defocusing distance. Hole spacing denotes the distance between adjacent micro holes formed by the picosecond Bessel beam on the front surface of the glass. Pulse energy signifies the energy level of each laser pulse employed in the cutting process. The defocusing distance refers to the position of the Bessel beam in relation to the glass surface during the cutting process. The 0 defocusing distance is defined as the position where the Bessel beam can generate uniform holes on the upper surface of the glass, and the value is positive when the Bessel beam moves upward. The cutting quality of the glass samples was assessed by analyzing the average surface roughness of the cross-sections. The impact of the processing parameters on the cutting quality was determined through the analysis of average surface roughness variations. While average surface roughness significantly affects edge strength, other factors can also contribute to its strength. Furthermore, in certain cases, the relationship between average surface roughness and edge strength is only moderately correlated [26]. Defects on large structures can have a greater negative impact on edge strength than average surface roughness [27]. This ultrafast laser Bessel beam cutting-assisted separation method can achieve superior edge strength compared to other reported processes, such as full laser ablation cutting [28]. Importantly, the average surface roughness measurement results remained relatively stable and were minimally affected by factors such as scratches, contamination, or measurement noise, thus ensuring a dependable evaluation of the cutting quality [29].

A three-factor, five-level orthogonal experiment was conducted to examine the impact of hole spacing, pulse energy, and defocusing distance on the cutting quality of the glass samples. The average surface roughness of the cross-sections served as the response variable for assessing the cutting quality. To assess the impact of each factor, a range of values was chosen for each based on upper and lower critical thresholds. Hole spacing was varied in the range of 4 μm to 12 μm. Values of hole spacing below 4 μm were found to cause overlapping micro-holes and excessive ablation on the front surface of the glass, resulting in perforation failure. Conversely, larger hole spacing values could lead to the deviation of crack propagation from the micro-hole path. The pulse energy percentage was set in the range of 100% to 80% to achieve the ablation of the 6 mm glass. Successful perforation required a minimum pulse energy percentage of around 80%. The actual pulse energy corresponding to the set power percentage was measured using a laser power meter. Defocusing distance varied in the range of 0 μm to 800 μm. A defocusing distance of 0 μm corresponded to the precise penetration of the Bessel beam into the front surface of the glass. Positive values indicated the upward movement of the focus. The depth of focus for the Bessel beam penetrating both the front and rear surfaces of the 6 mm glass was measured to be 7.4 mm at full power. Other parameters in the experiment were held constant. The factor levels of the orthogonal experiment are summarized in Table 1. Parameter optimization was performed using the SPSS 26.0 software [30].

## 3. Results and Discussion

### 3.1. Variance Analysis of the L25 (5^3^) Orthogonal Experiments

**Figure 2:**
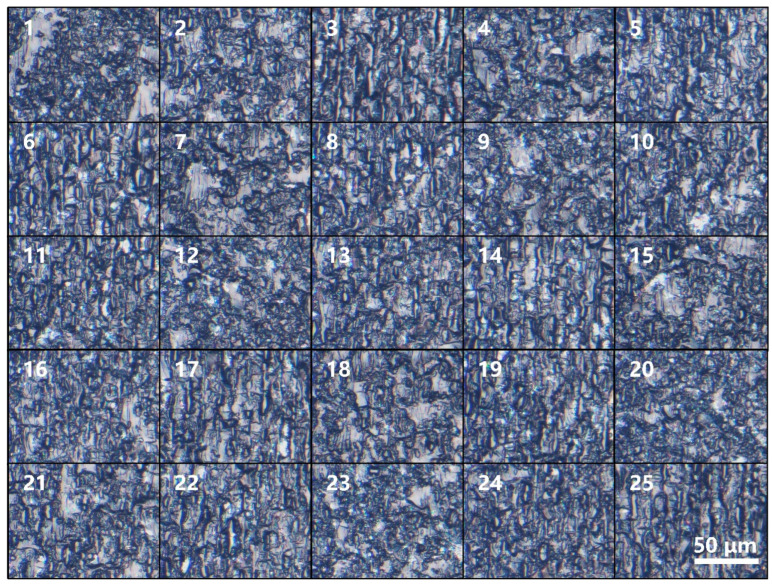
Typical microscope image of each sample.

Average surface roughness measurements were obtained at three positions: near the front surface, a rear surface, and the middle of the cross-section of each glass sample to assess the cutting quality. The average surface roughness at these three positions was used as the response variable for the orthogonal experiment. The orthogonal experiment was designed with 25 different working conditions, and the corresponding experimental results for average surface roughness are shown in Table 2. Figure 2 illustrates the typical cross-sectional morphologies for each parameter setting.

**Figure 3:**
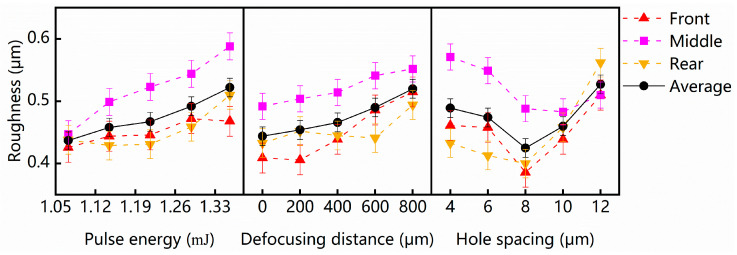
Estimated marginal means of the effect of influence parameters on the average surface roughness of glass-cutting sample cross-sections.

The average surface roughness of the cross-section was significantly influenced by all three parameters: hole spacing, pulse energy, and defocusing distance (*p* < 0.05). The order of significance, in terms of their influence on the average surface roughness of the cross-section, was determined to be as follows: hole spacing, pulse energy, and defocusing distance. The statistical analysis conducted in this study determined the optimal levels for each individual factor, as well as the optimal combination of multiple factors in the orthogonal experiment. The results of the statistical analysis for each parameter are presented in Table 3 and Figure 3. The analysis revealed a decrease in the average surface roughness of the cross-section as the pulse energy decreased within the range of 1.072 mJ to 1.356 mJ. The average surface roughness increased as the defocusing distance increased. Increasing the hole spacing from 4 μm to 12 μm initially decreased the average surface roughness of the cross-section, reaching its minimum at 8 μm hole spacing before it then increased. The optimum processing parameters for the ablation cutting of 6 mm thick soda-lime glass using a picosecond laser Bessel beam were determined based on the statistical analysis results. The best average surface roughness of the cross-section was achieved with a hole spacing of 8 μm, pulse energy of 1.072 mJ, and a defocusing distance of 0 μm, as shown in Table 3.

### 3.2. The Influence of Processing Parameters

Laser ablation significantly affects the quality of the cutting surface, encompassing the decomposition of the processed material, the heat-affected zone, the formation of microcracks, and the roughness of the cross-section. Moreover, the spacing between the holes ablated by the picosecond laser influenced the formation of the crack propagation path, thereby affecting the roughness of the cross-section. Generally, smaller spacing between micro holes promotes crack propagation, although it does not guarantee an enhanced quality cutting surface [22]. If the spacing between the holes becomes smaller than the hole diameter, the heat-affected zone and debris from the preceding hole can disrupt the ablation of the subsequent hole, leading to processing failures due to some micro holes being unable to penetrate thick glass. Conversely, if the hole spacing is too large, crack propagation cannot form a continuous path along the micro holes.

When the hole spacing is too small, a large number of connection interferences between holes are easily generated, and crack growth is uncontrollable. When the hole spacing is too large, the crack expansion process is difficult to connect together due to the lack of weak zones. Based on the statistical analysis results presented in Figure 3, it is evident that increasing the hole spacing from 4 μm to 12 μm initially reduces the average surface roughness of the cross-section, reaching a minimum at 8 μm hole spacing before subsequently increasing. The trends of surface roughness observed at the three different positions (both sides and middle) aligned with the average surface roughness. Despite the Bessel beam’s relatively long energy deposition length, its energy intensity distribution along the laser propagation direction is non-uniform. The energy distribution of the Bessel beam rapidly increases to a peak and then gradually decreases. The non-uniformity of the energy intensity distribution leads to the irregular morphology of the glass perforation, consequently affecting the roughness of the cross-section after separation. The energy applied to both sides of the cross-section is relatively low, leading to a smaller overlapped ablation area and reduced interference. When increasing hole spacing, the wider gap between the holes hinders the formation of complete and uniform cracks, resulting in a rougher cross-section on both sides. The middle section of the cross-section experiences the highest energy intensity from the Bessel beam, resulting in greater surface roughness compared to the sides.

Crack propagation along the path of the micro holes was facilitated by scanning a CO_2_ laser, resulting in the formation of cleavage surfaces on the glass samples. Small hole spacing resulted in overlapped ablation, causing a more extensive removal of material. Consequently, the crack propagation became uncontrollable, resulting in increased roughness of the cross-sections. Increasing the hole spacing resulted in the generation of micro holes and microcracks through laser ablation. With larger hole spacing, the amount of ablation on the cross-section decreased, but the occurrence of brittle damage during the separation process increased. With larger hole spacing, the amount of ablation on the cross-section decreased, but the occurrence of brittle damage during the separation process increased. In summary, the micro-morphology observed in the cross-sections varied depending on the hole spacing. Smaller hole spacing resulted in more extensive ablation and overlap damage, while larger hole spacing led to reduced ablation and increased brittle damage during separation. However, optimizing the hole spacing to a suitable value prevented edge chipping after separation, indicating better control over crack propagation and the improved smoothness of the resulting cross-section.

The insufficient ablation of certain micro holes in the glass may occur when the pulse energy is relatively low. This prevention of micro holes from penetrating both the front and rear surfaces of the glass results in increased roughness along the cross-section after the separation process. Conversely, when the laser power is relatively high, excessive ablation of the micro holes can occur. As a result, the adhesion and melting of the glass surrounding the micro holes occur, leading to an uneven distribution of stress. Moreover, these defects exacerbate the complexity of the separation process and contribute to increased roughness along the cross-section.

High-laser power tends to create larger hole diameters and also larger ablation zones. A low-laser power can lead to crack expansion processes that require greater thermal stresses or even prevent the realization of lobes due to incomplete ablation. Figure 3 illustrates the impact of pulse energy on the average surface roughness of the cross-section. It is evident that a pulse energy of 1.072 mJ yields the most favorable outcomes in minimizing average surface roughness. A decrease in pulse energy within the range of 1.072 mJ to 1.356 mJ corresponds to a decrease in the average surface roughness of the cross-section. This can be attributed to the relatively high energy intensity in the middle section, which promotes overlapped ablation. Employing lower pulse energy leads to a reduction in the ablated area, resulting in finer cutting quality, particularly in the middle section. However, the alteration in surface roughness at the sides of the cross-section is less significant at low pulse energy. This suggests that the energy deposition on both sides is already sufficiently low within the range of 1.072–1.216 mJ.

Although the regular pattern was not clearly evident, the influence of the defocusing distance on the average surface roughness of the cross-section was indeed significant. A change in the defocusing distance of 200 μm may be inadequate when compared to the 8 mm Bessel zone. The peak energy deposition of the Bessel beam consistently occurred within the 6 mm thickness of the glass, with a relatively small variation in the minimum energy affecting the glass. As a result, the impact on the morphology in the middle of the micro holes was minimal. The size of the hole can serve as an indication of the actual energy applied to the glass material. Therefore, the roughness of the cross-section is minimized when there is a minimal energy difference between the maximum and minimum energy required to perforate the glass. The key concept for optimizing the defocusing distance should involve reducing the energy disparity between the front and rear surfaces.

Despite the intuitive expectation that positioning the top of the energy deposition distribution closer to the front surface of the glass can minimize the energy difference, the lowest average surface roughness of the cross-section was actually achieved at a defocusing distance of 0 μm, as demonstrated in Figure 3. A defocusing distance of 0 μm implies that the laser forms ablated holes precisely at the front surface, leading to minimal surface roughness. During practical processing, the laser requires additional energy as it propagates from the front surface to the rear surface of the glass. Moreover, the refractive index of the glass affects the intensity distribution of the Bessel beam within the glass. Despite the decreasing surface roughness at the rear side as the Bessel beam moves upward, achieving an equivalent level of surface roughness near the rear surface, compared to the front surface, at a defocusing distance of 0 μm remains challenging. Thus, to minimize the energy difference and achieve superior roughness results, the optimal top position of the energy deposition distribution should be closer to the rear surface compared to the theoretical energy distribution of the Bessel beam.

**Figure 4:**
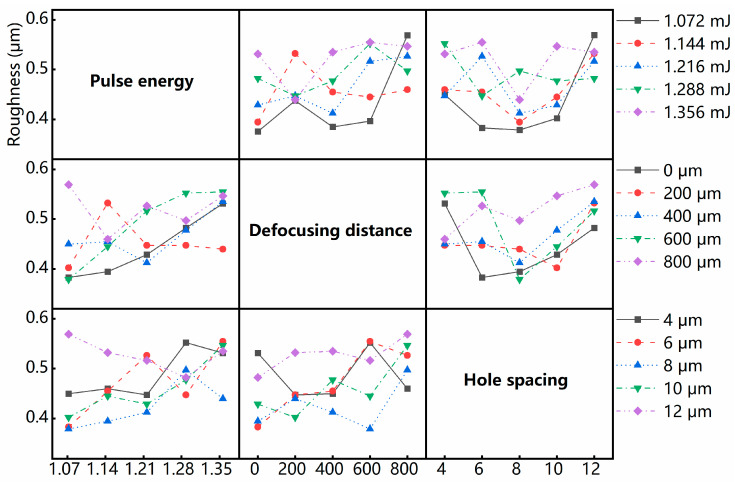
Interaction between each parameter for average surface roughness.

The interaction graph obtained through interaction analysis can show how the relationship between a parameter and the evaluation index depends on the value of the second parameter. Figure 4 shows the interaction between each parameter of average surface roughness. The interaction analysis between the process parameters corresponding to the optimal average surface roughness (1.072 mJ–0 μm–8 μm) shows that (1) when the pulse energy is 1.072 mJ, the interaction between the pulse energy and defocusing distance is smaller than other pulse energy levels, and the interaction is the smallest when the defocusing distance is 0 μm. (2) When the hole spacing is 8 μm, the interaction between the defocusing distance and hole spacing is smaller than other defocusing distance levels, and the interaction is relatively small when the defocusing distance is 0 μm. (3) When the hole spacing is 8 μm, the interaction between hole spacing and pulse energy is smaller than the other hole spacing levels, and the interaction is the smallest when the pulse energy level is 1.072 mJ.

### 3.3. Optimal Processing Parameters

**Figure 5:**
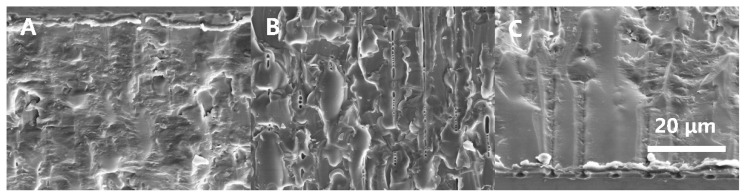
SEM images of the cross-section of the optimal sample (**A**) near the front surface, (**B**) the middle, and (**C**) near the rear surface.

The range of analysis results of the orthogonal experiment indicated that the influence of each parameter on the average surface roughness was similar, and the optimal parameter combination of 1.072 mJ–0 µm–8 µm yielded the lowest average surface roughness. The average surface roughness at these parameter levels was 0.343 µm, surpassing the other results obtained in the orthogonal experiment. Additionally, the glass-cutting edge exhibited high quality with minimal edge chipping. The SEM morphology analysis of the cutting surface near the front, middle, and rear surfaces is presented in Figure 5A, Figure 5B, and Figure 5C, respectively. This analysis revealed the absence of noticeable defects on the glass sample’s cross-section, indicating that the cutting process employing the optimal parameter combination achieved a satisfactory surface quality, free from significant imperfections or irregularities.

**Figure 6:**
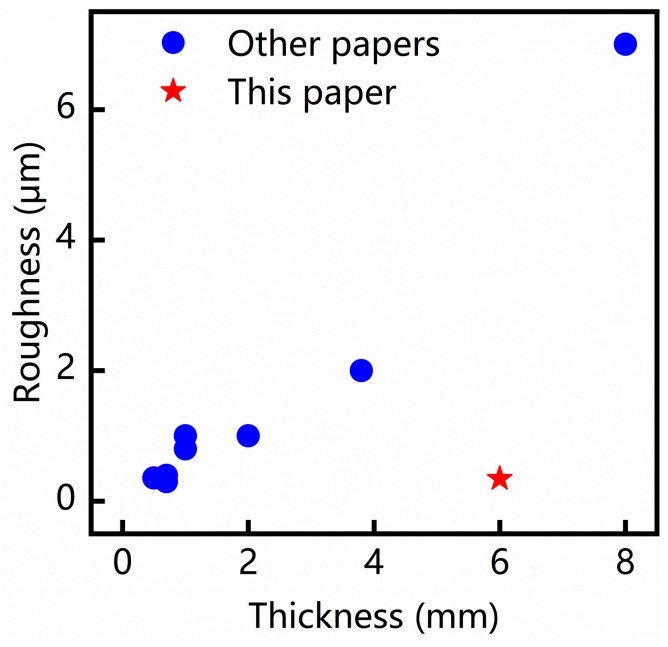
Demonstrated roughness against thickness of glass cutting utilizing pulsed Bessel beams [9,13,14,26,27,28,29].

Consequently, the statistical analysis of the estimated marginal means, taking into account the combined effect of the three optimal parameters (hole spacing of 8 μm, pulse energy of 1.072 mJ, and defocusing distance of 0 μm), revealed that the cross-sectional average surface roughness reached a minimum value of 0.343 μm. This finding suggests that the chosen parameter combination yielded the highest surface quality for the glass-cutting process. As observed in Table 4, achieving a high-quality cutting surface becomes increasingly challenging as the glass thickness increases. In contrast, the surface roughness of the glass-cutting samples in this study is superior and comparable to that of thin glass-cutting samples within the 1 mm range, as illustrated in Figure 6.

## 4. Conclusions

In this research, low average surface roughness cutting (0.343 μm) of 6 mm thick soda-lime glass was achieved for the first time using ultrafast laser perforation assisted by CO_2_ laser separation. The burst mode and PSO function of the laser source facilitate the formation of uniform and closely spaced micro holes in the glass. Following the scanning of a CO_2_ laser, the glass is separated along the direction of these micro holes. The morphology of the cross-sections, which directly affects the roughness, is greatly influenced by the pulse energy and hole spacing. The laser power affects the cross-section morphology and, thus, the roughness by influencing the pore size and the ablation zone. Hole spacing affects the cross-section morphology and, thus, the roughness by influencing the crack propagation process. The defocusing distance impacts the morphology on both sides of the cross-sections, thereby influencing the roughness. Through an orthogonal experiment, the optimal processing parameters to achieve the minimum average surface roughness in cutting 6 mm soda-lime glass were determined. These parameters consist of a hole spacing of 8 μm, a pulse energy of 1.072 mJ, and a defocusing distance of 0 μm. Statistical analysis indicates that the order of influence of the three factors is as follows: hole spacing, pulse energy, and defocusing distance. By utilizing this method and the optimal cutting parameters, it was possible to achieve high surface quality while maintaining a cutting speed exceeding 200 mm/s. The average surface roughness of the cutting surface can be reduced to 0.343 μm. Additionally, this cutting method enables the cutting of glass samples with curved profiles and is also applicable to thicker glass.

## Figures and Tables

**Table 1:** Picosecond Bessel laser cutting process parameters and levels.

Process Parameters	Unit	Factor Level				
		1	2	3	4	5
Pulse energy	(mJ)	1.356	1.288	1.216	1.144	1.072
Defocusing distance	(μm)	0	200	400	600	800
Hole spacing	(μm)	4	6	8	10	12

**Table 2:** L25 (5^3^) orthogonal experiments and experimental results.

No.	Pulse Energy (mJ)	Defocusing Distance (μm)	Hole Spacing (μm)	Average Surface Roughness (μm)
1	1.356	0	4	0.532
2	1.356	200	8	0.440
3	1.356	400	12	0.535
4	1.356	600	6	0.555
5	1.356	800	10	0.547
6	1.288	0	12	0.483
7	1.288	200	6	0.448
8	1.288	400	10	0.478
9	1.288	600	4	0.553
10	1.288	800	8	0.498
11	1.216	0	10	0.429
12	1.216	200	4	0.448
13	1.216	400	8	0.413
14	1.216	600	12	0.517
15	1.216	800	6	0.527
16	1.144	0	8	0.395
17	1.144	200	12	0.533
18	1.144	400	6	0.455
19	1.144	600	10	0.445
20	1.144	800	4	0.460
21	1.072	0	6	0.383
22	1.072	200	10	0.403
23	1.072	400	4	0.450
24	1.072	600	8	0.379
25	1.072	800	12	0.569

**Table 3:** Range analysis of the influence parameters.

Dependent Variable	Value	Mean Surface Roughness (μm)	Standard Error	95% Confidence Interval	
				Lower Bound	Upper Bound
Pulse energy (mJ)	1.072	0.437	0.015	0.404	0.47
	1.144	0.458	0.015	0.425	0.49
	1.216	0.467	0.015	0.434	0.5
	1.288	0.492	0.015	0.459	0.525
	1.356	0.522	0.015	0.489	0.555
Defocusing distance (μm)	0	0.444	0.015	0.412	0.477
	200	0.454	0.015	0.422	0.487
	400	0.466	0.015	0.433	0.499
	600	0.49	0.015	0.457	0.523
	800	0.52	0.015	0.487	0.553
Hole spacing (μm)	4	0.489	0.015	0.456	0.521
	6	0.474	0.015	0.441	0.506
	8	0.425	0.015	0.392	0.458
	10	0.46	0.015	0.428	0.493
	12	0.527	0.015	0.495	0.56

**Table 4:** Demonstrated glass-cutting utilizing pulsed Bessel beams.

Thickness (mm)		Surface Roughness (μm)	Source
0.5	Silica glass	0.355	K. Liao et al. [13]
0.7	Chemically strengthened glass	0.395	Z. Yang et al. [31]
0.7	Tempered glass	0.3	M.K. Bhuyan et al. [32]
1	Soda-lime glass	1.0	J. Dudutis et al. [9]
2	Silicate glass	0.8	M. Jenne et al. [33]
3.8	Float glass	2.30	T. Dietz et al. [34]
8	Soda-lime glass	7.0	A. Feuer et al. [14]
6	Soda-lime glass	0.343	This paper

## Data Availability

The original contributions presented in the study are included in the article, further inquiries can be directed to the corresponding author.
